# Repercussion of the implementation of the Picture Exchange Communication System - PECS in the overload index of mothers of children with Autism Spectrum Disorder

**DOI:** 10.1590/2317-1782/20212021109

**Published:** 2022-01-07

**Authors:** Carine Ferreira, Sheila Cavalcante Caetano, Jacy Perissinoto, Ana Carina Tamanaha

**Affiliations:** 1 Departamento de Fonoaudiologia, Universidade Federal de São Paulo – UNIFESP - São Paulo (SP), Brasil.; 2 Departamento de Psiquiatria, Universidade Federal de São Paulo – UNIFESP - São Paulo (SP), Brasil.

**Keywords:** Autism Spectrum Disorder, Mothers, Caregivers, Communication, Speech Language Hearing Sciences

## Abstract

**Purpose:**

The aim of this study was to analyze the repercussion of the implementation of PECS on the burden index of mothers of children with Autism Spectrum Disorder (ASD).

**Methods:**

This was a longitudinal study (CEP 0403/2017). The sample consisted of 20 mother and child with ASD. The mothers were on average 41 years and 5 months and the children were 7 years and 2 months old. Fifteen children were male and five were female. The brazilian version of the Burden Interview scale was applied to verify the level of caregiver burden. The Autism Behavior Checklist (ABC) was applied to the analysis of non-adaptive behaviors and to analyze the lexical repertoire: Auditory and Expressive Vocabulary Tests. The PECS Implementation Program was composed of 24 sessions of individual speech therapy with the active presence of mothers. At the end all children and mothers were reevaluated with part of the instruments.

**Results:**

There was a tendency to reduce maternal overload indexes after the implementation of the PECS. There was a significant decrease in non-adaptive behaviors and an increase in the expressive and auditory vocabulary indexes of the children at the final moment of the study. We did not observe a significant correlation between the degrees of overload with age, schooling and intellectual quotient of children, nor schooling and maternal socioeconomic status.

**Conclusion:**

It was possible to analyze the repercussion of the implementation of the PECS on the burden of mothers of children with ASD assisted by the Program.

## INTRODUCTION

The burden of caregivers of people with chronic pathologies is described as a disturbance resulting from dealing with the physical dependence and mental incapacity of the individual receiving attention and care. By assuming the role of guardian, caregiver or responsible for the well-being and provision of care to a dependent family member, the individual is subject to tension and stressors, but also to gains, such as feeling satisfaction and well-being for what he/she can provide your family member^(1)^.

The initial impact of a diagnosis can be overwhelming for families, requiring a long period of adaptation and balance. In the case of the diagnosis of Autism Spectrum Disorder (ASD), this reality is no different.

In fact, the ASD is characterized by serious and chronic impediments in the areas of interaction and social communication, and by a restricted and stereotyped repertoire of interests^(2,3)^.

The developmental impairment presented by children affected by this condition can have several implications for family dynamics, from physical and mental overload resulting from daily life attributions, high levels of stress and low quality of life index for their families, to the possibility of developing adaptive capacity and resilience. These aspects can interfere in personal, family, professional and social life; and can predispose the caregiver to conflicts^(1,4-6)^.

Faced with such an invasive and serious condition, the family dynamics undergoes mobilizations that range from aspects related to the physical, psychological, social and even financial quality of life of direct caregivers^(1,4-6)^. Parents have to mourn the loss of the ideal child so that they can perceive their child's real capabilities and potential^(6)^.

Studies indicate that the adherence of families to specialized treatment has a direct impact on the child's development and can improve family dynamics by reducing the burden of all involved^(1,3-6)^.

The speech-language therapy work in a language with the child with ASD presupposes the participation of the family, from the assessment to the selection and conduction of intervention resources. Alternative and augmentative communication systems, such as the Picture Exchange Communication System (PECS), provide an effective means of allowing children with severely limited communication skills to exercise control over the environment by requesting preferred items^(7-12)^.

Parent training in its implementation has shown benefits for children with ASD, improving their communicative responses. Other studies also report an increase in the number of vocalizations with communicative intent or functional speech, acquisitions that are fundamental for the development of human communication^(9-12)^.

The hypothesis we consider is that the guided participation of parents in speech-language therapy intervention with an alternative communication system reduces the burden as it expands the child's communication resources.

This study aimed to analyze the impact of PECS implementation on the burden rate of mothers of children with ASD.

And as specific objectives: to verify the effect of using PECS on non-adaptive behavior and auditory and expressive vocabulary indexes. And finally, to correlate the initial and final maternal burden indexes to age group, education, children's intellectual quotient, and maternal education and socioeconomic level.

## METHODS

This is a longitudinal study. All parents were aware of the study procedures and signed the Informed Consent Form (ICF), in accordance with the suggestions of the Institution's Research Ethics Committee (CEP 0403/2017).

### Sample

A convenience sample was constituted, consisting of 20 mother-child dyads with ASD, attended at the Center for Speech-Language Pathology Investigation of Children and Adolescents in Autism Spectrum Disorder (*Núcleo de Investigação Fonoaudiológica em Crianças e Adolescentes com Transtorno do Especto Autista* (NIFLINC TEA)), Department of Speech-Language Pathology, at “Universidade Federal de São Paulo” Federal University of São Paulo (UNIFESP), in the period from March 2016 to March 2019.

The mothers had an average of 41 years and 5 months (SD=7.9). Twelve of them had completed higher education (60%) and eight (40%) had completed high school.

Regarding the socioeconomic level of the families, eight (40%) belonged to class A/B (high) and twelve (60%) to classes C/D (medium-low), according to the ABEP's socioeconomic classification^(13)^.

Of the total number of children, fifteen were male (75%) and five female (25%); aged between 6 and 12 years old (average = 7 years and 2 months), treated and diagnosed with ASD by a multidisciplinary team, according to the diagnostic criteria of the DSM-5^(2)^.

Regarding the speech extension obtained from the application of the Vocal Behavior Assessment^(14)^, 18 children (90%) demonstrated non-verbal production (babble emission and/or vocalizations) and two children (10%), minimal verbal production: emission of isolated words or juxtaposition (without the use of verbs), during the speech-language evaluation period.

Regarding the cognitive profile, the distribution of Intellectual Quotient (IQ) values ​​was concentrated in the lower range, with an average score equal to 50.5 (SD=9.4)^(15)^.

All children were regularly enrolled in regular schools due to the Brazilian policy of school inclusion, on average for forty-five months (SD=21.9) and had already been exposed to previous speech-language therapy intervention in different care services, for at least six months of duration, to guarantee that the communicative profile was characterized as non-verbal or by minimal verbalization.

As inclusion criteria, it was considered: the diagnosis of ASD; the age group; the absence of verbal communication or minimal verbalization; the child's attachment to educational institutions and the family's availability to participate in speech-language therapy sessions with a minimum adherence of 75%.

As exclusion criteria, it could be considered the presence of neurological alterations (structural and/or functional impairment of the Central Nervous System (CNS)), malformations and/or known genetic syndromes, physical, hearing, visual and/or motor impairments.

### Procedures

To assess maternal burden, the Brazilian version of the Burden Interview Scale^(16)^ was applied, whose objective is to verify the level of burden of caregivers of individuals with mental and physical disabilities, through interviews.

The scale includes 22 questions covering the areas of health, social and individual life, financial situation, emotional stability and interpersonal relationships. These questions reflect how people feel about taking care of another person. The questions referring to items 1 to 21 are scored as: 0 - never; 1 - rarely; 2 - sometimes; 3 - frequently; 4 - always. Question 22 is general and in it, the respondent is asked to assess how much he/she considers to be overloaded due to their role. In this question, the possible answers correspond: 0 - not at all; 1- a little; 2 - moderately; 3 - a lot and 4 - extremely. After all, questions are scored and summed, the final score is obtained. The higher the final score, the greater the caregiver burden, being: < 21 points - no burden; from 21 to 40 points - moderate overload; 41 to 60 points - variation between moderate to severe overload and > or equal to 61 - severe overload.

For the analysis of non-adaptive behaviors, the Autism Behavior Checklist (ABC)^(14)^ was applied as an interview, which consists of a list of 57 non-adaptive behaviors divided into five areas: sensory; use of the body and object; relational, language and personal-social.

And to assess the vocabulary indices, the following were applied:

- Auditory Vocabulary Test^(17)^, whose objective was to assess the receptive vocabulary through the identification of pictures.

- Expressive Vocabulary Test^(17)^, whose objective was to assess the child's expressive vocabulary through picture naming.

### The PECS Implementation Program

The program consisted of 24 individual speech-language therapy sessions with the presence of the mothers. Each session lasted 45 minutes and was held weekly. All Speech-Language Pathologists involved were professionals trained and certified in PECS. All sessions were filmed so that the children's behaviors were recorded in the progress monitoring protocols in each phase, as proposed in the PECS Training Manual^(7)^. The records were made by researchers who were not involved in the direct care of the children.

After completion of the Program, all children and their respective mothers were reassessed with part of the instruments used in the initial phase of the study.

### Statistical method

Initially, a descriptive analysis of the data was performed. The Paired T-Test or Wilcoxon Test was used for comparative analysis of the variables: maternal overload; ABC indexes and the children's auditory and expressive vocabulary, in the two moments of the study. To analyze the correlations between the initial and final maternal burden indexes with: age, education, child's IQ; and the education and socioeconomic level of the mothers, the Spearman Correlation Coefficient was used. A significance level of 0.05% was considered.

## RESULTS

Table 1 shows the frequency of the degrees of maternal burden in the two moments of the study.

**Table 1:** Frequency of the degrees of maternal burden at the two moments

Time /	Pre-PECS		Post-PECS	
Degree of overload	N	%	N	%
Absent	0	0%	1	5%
Mild	0	0%	0	0%
Moderate	12	60%	15	75%
Moderate Severe	6	30%	3	15%
Severe	2	10%	1	5%
Total	20	100%	20	100%

Caption: N = number of mothers

Table 2 shows the comparisons between maternal overload, ABC indexes and children's auditory and expressive vocabularies, in the two moments of the study.

**Table 2:** Comparative analysis between maternal burden, ABC indexes and children's auditory and expressive vocabulary, in the two moments of the study

		Pre	Post	T-paired test or Wilcoxon (*p*)	Results
Overload	Average	43.0	39.0		
	Median	41.0	40.0	0.224	Pre = Post
	Standard deviation	13.0	12.1		
	N	20	20		
ABC SE	Average	13.2	9.1		
	Median	13.5	9.5	0.001^*^	Pre > Post
	Standard deviation	4.7	4.5		
	N	20	20		
ABC RE	Average	22.6	17.5		
	Median	22.0	18.5	<0.001*	Pre > Post
	Standard deviation	6.8	6.4		
	N	20	20		
ABC CO	Average	20.0	16.8		
	Median	20.0	17.0	0.093*	Pre > Post
	Standard deviation	7.9	7.8		
	N	20	20		
ABC LG	Average	13.3	10.5		
	Median	12.5	9.0	0.088	Pre = Post
	Standard deviation	4.8	4.9		
	N	20	20		
ABC PS	Average	16.8	10.9		
	Median	18.5	10.5	<0.001*	Pre > Post
	Standard deviation	4.1	5.2		
	N	20	20		
ABC Total	Average	85.9	64.0		
	Median	86.0	59.5	<0.001*	Pre > Post
	Standard deviation	16.8	15.0		
	N	20	20		
Auditory Voc	Average	3.0	7.4		
	Median	0.0	0.0	0.008*	Pre < Post
	Standard deviation	8.0	12.6		
	N	20	20		
Expressive Voc	Average	2.7	4.2		
	Median	0.0	0.0	0.018*	Pre < Post
	Standard deviation	8.8	9.5		
		20	20		

* Statistical significance

Caption: ABC SE = Autism Behavior Checklist Sensory; ABC RE = Autism Behavior Checklist Relational; ABC CO = Autism Behavior Checklist Use of Body and Object; ABC LG = Autism Behavior Checklist Language; ABC PS = Autism Behavior Checklist Personal Social; Voc = Vocabulary; N = Number of children

In Table 3, it can observe the correlations between the initial and final maternal burden indexes with the variables age, schooling time and the child's IQ; and time of maternal education and socioeconomic level of the family.

**Table 3:** Correlation between the initial and final maternal burden indexes with the variables: age, educational level and child's IQ; time of maternal education and socioeconomic level

				Overload 1	Overload 2
Age	Correlation Coefficient			- 0.315	- 0.042
	*p*-value			0.176	0.860
	N			20	20
Child Schooling	Correlation Coefficient			0.392	- 0.124
	*p*-value			0.087	0.601
	N			20	20
IQ	Correlation Coefficient			- 0.164	- 0137
	*p*-value			0.491	0.566
	N			20	20
Maternal Education	Correlation Coefficient			0.002	- 0.267
	*p*-value			0.994	0.256
	N			20	20
SEL	Correlation Coefficient			- 0.099	- 0.132
	*p*-value			0.688	0.589
	N			20	20

Caption: N = number of children; IQ = Intellectual Quotient; SEL = Socioeconomic level

## DISCUSSION

We noted that at the beginning of the study, all mothers reported some degree of burden, with a predominance of mention of the moderate degree. These results confirm that mothers of children with ASD are highly vulnerable to emotional overload and situations of parental stress, as the care for their children's well-being commonly falls on them^(1,3-6)^.

And although there was no statistical significance in the comparative analysis of the mean burden indices at the two moments of the study (*p*=0.224), we noticed a tendency towards a reduction in values ​​after the implementation of the PECS Program. There was a slight decrease in the degrees of severe, moderately-severe overload and mention was made of the absence of overload in one of the cases, in the second stage of the study. These results highlighted the importance of the active participation of families in the treatment^(18)^. There is a consensus that parental empowerment is a major factor in facing the challenges that ASD imposes on all family members^(18-22)^.

Another relevant point that must be considered here refers to the appropriation of the alternative and augmentative communication system that must have allowed more efficient communicative exchanges between children and their mothers. Thus, by encouraging and encouraging children to demonstrate their wishes and intentions through the cards, the PECS system improved the quality of communication and, consequently, may have influenced the reduction in the perception of emotional burden on the part of mothers throughout the program^(9-12,18-22)^.

In the comparative analysis between the two moments of the study, we verified that there was a tendency towards a reduction in non-adaptive behaviors observed in all areas of the ABC. There was statistical significance in the Sensory (*p*=0.001), Relational (*p*=0.001), Use of Body and Object (*p*=0.093) and Personal-Social (*p*=0.001) areas, as well as in the Total values ​​(*p*=0.001). This decrease in behavioral atypia, observed from the perspective of mothers, confirmed the positive impact of using PECS, not only enabling more efficient communicative exchanges, but also improving the quality of social interactions. Even in the area of Language (*p*=0.088) in which there was no statistical significance, the index in the second moment of the study showed a downward trend. By adding visual cues to auditory-verbal information, PECS expanded verbal comprehension and positively impacted communicative exchanges and social engagement^(9-12,23-27)^.

We also observed a significant increase in responses both in testing expressive vocabulary (*p*=0.018) and auditory (*p*=0.008). The improvement in communicative performance, both its expressive and receptive character, corroborates the positive effect of PECS in increasing verbalization and, above all, in the verbal comprehension of its users, as described by different studies (7-12,23-27).

No significant correlations were identified between the initial and final maternal burden indexes and the variables age (*p*=0.176; *p*=0.860), education (*p*=0.087; *p*=0.601) and intellectual quotient of children (*p*=0.491; *p*=0.566). In other words, the age group, the average length of schooling and the intellectual quotient of the children did not influence the mothers' perception of their burden. The same occurred in relation to the time of maternal education (*p*=0.994; *p*=0.256) and the families' socioeconomic level (*p*=0.688; *p*=0.589).

Therefore, maternal overload should be much more linked to concerns inherent to the awareness that ASD is a severe and lifelong condition, which affects families deeply regardless of the child's age, cognitive potential or stage of development^(18-24)^.

And even though we are aware that the financial condition of families can be a barrier to accessing specialized services, which can lead to an increase in parental stress^(21)^, in this study we did not find a correlation between socioeconomic level and maternal burden. Perhaps because the families are linked to a reference service in the treatment of ASD, this has alleviated the perception of mothers' burden in the interviews.

Caring for parents, sometimes providing them with accurate information about the child's development, receiving questions and understanding requests, sometimes inviting them to participate as agents in the language process is a fundamental task in the child's speech-language therapy care^(19)^.

The results of this study lead us to reflect on the importance of the Speech-Language Pathologist’s attentive look also for families, especially for the main caregiver. People with ASD demand care and knowing the emotional state of this caregiver, we can improve and more accurately target our therapeutic approaches^(21-30)^.

### Limitations

We are aware that the sample size must be considered a limitation of the study as it may have reduced the potential of statistical treatment. Therefore, we encourage further studies on the effects of PECS with larger samples, longer exposure periods, and double-blind clinical trials to be conducted. In addition, we strongly recommend that future studies consider evaluating symptoms of maternal anxiety and depression.

## CONCLUSION

There was a trend towards a reduction in maternal burden rates after the implementation of the PECS. There was a significant decrease in non-adaptive behaviors and an increase in the children's expressive and auditory vocabulary indices at the end of the study. We did not observe a significant correlation between the degrees of burden with the children's age, education and intellectual quotient; nor with maternal education and socioeconomic status.

Thus, we were able to analyze the impact of PECS implementation on the burden of mothers of children with ASD assisted by the Program.

## References

[1] Misquiatti ARN, Brito MC, Ferreira FTS, Assumpção FB (2015). Sobrecarga familiar e crianças com Transtornos do Espectro do Autismo: perspectiva dos cuidadores. Rev CEFAC.

[2] APA: American Psychiatric Association (2014). Manual Diagnóstico e Estatistico de Transtornos Mentais – DSM 5..

[3] Klin A, Jones W (2018). An agenda for 21st century neurodevelopmental medicine: lessons from Autism. Rev Neurol.

[4] Hong J, Bishop-Fitzpatrick L, Smith LE, Greenberg JS, Mailick MR (2016). Factors associated with subjective quality of life of adults with autism spectrum disorder: self-report versus maternal reports. J Autism Dev Disord.

[5] Jones S, Bremer E, Lloyd M (2017). Autism spectrum disorder: family quality of life while waiting for intervention services. Qual Life Res.

[6] Reed P, Sejunaite K, Osborne LA (2016). Relating between self-reported health and stress in mother of children with Autism Spectrum Disorder. J Autism Dev Disord.

[7] Bondy A, Frost L (2009). Manual de treinamento do sistema de comunicação por troca de figuras..

[8] Ferreira C, Bevilacqua M, Ishihara M, Fiori A, Armonia A, Perissinoto J (2017). Selection of words for implementation of the Picture Exchange Communication System - PECS in non-verbal autistic children. CoDAS.

[9] Doherty A, Bracken M, Gormley L (2018). Teaching children with autism to initiate and respond to peer mands using Picture Exchange Communication System. Behav Anal Pract.

[10] Landa R, Hanley GP (2016). An evaluation of multiple-schedule variations to reduce high-rate requests in the picture exchange communication system. J Appl Behav Anal.

[11] Agius MM, Vance M (2016). A comparison of PECS and Ipad to teach requesting to preschollers with autistic spectrum disorders. Augment Altern Commun.

[12] Thiemann-Bourque K, Brady N, McGuff S, Stump K, Naylor A (2016). Picture Exchange Communication System and Pals: a peer-mediated augmentative and alternative communication intervention for minimally verbal preschoolers with autism. J Speech Lang Hear Res.

[13] ABEP: Associação Brasileira de Empresas de Pesquisa (2018). Critério de Classificação Econômica Brasil..

[14] Marteleto MRF, Pedromônico MR (2005). Validity of Autism Behavior Checklist (ABC): preliminary study. Rev Bras Psiquiatr.

[15] Weschler D (2002). WISC III Escala de inteligência para crianças..

[16] Scazufca M (2002). Versão brasileira da escala *Burden Interview* para avaliação da sobrecarga do cuidado em cuidadores de pessoas com doenças mentais. Rev Bras Psiquiatr.

[17] Capovilla FC, Negrão VB, Damazio M (2011). Teste de Vocabulário Auditivo e Teste de Vocabulário Expressivo..

[18] Siah PC, Tan SH (2016). Relationships between sense of coherence, coping strategies and quality of life of parents of children with Autism in Malaysia: a study of chinese parents. Disabil CBR Incl Dev.

[19] Picardi A, Gigantesco A, Tarolla E, Stoppioni V, Cerbo R, Cremonte M (2018). Parental burden and its correlates in families of children with Autism Spectrum Disorder: a multicentre study with two comparison groups. Clin Pract Epidemiol Ment Health.

[20] Bozkurt G, Uysal G, Düzkaya DS (2019). Examination of care burden and stress coping styles of parents of children with Autism Spectrum Disorder. J Pediatr Nurs.

[21] Ribeiro SH, Paula CS, Bordini D, Mari JJ, Caetano SC (2017). Barriers to early identification of autism in Brazil. Rev Bras Psiquiatr.

[22] Moretto G, Ishihara MK, Ribeiro M, Caetano SC, Perissinoto J, Tamanaha AC (2020). Interferência do meio comunicativo da criança com Transtorno do Espectro do Autismo na qualidade de vida de suas mães. CoDAS.

[23] Haney JL, Houser L, Cullen JA (2018). Parental perceptions and child emotional and behavioral problems in Autism. J Autism Dev Disord.

[24] Siller M, Hotez E, Swanson M, Delavenne A, Hutman T, Sigman M (2018). Parent coaching increases the parents´ capacity for reflection and self-evaluation: results from a clinical trial in autism. Attach Hum Dev.

[25] Gilroy SP, Leader G, McCleery JP (2018). A pilot community-based randomized comparison of speech generating devices and the PECS for children diagnosed with Autism Spectrum Disorder. Autism Res.

[26] Brignell A, Chenausky KV, Song H, Zhu J, Suo C, Morgan AT (2018). Communication interventions for Autism Spectrum Disorder in minimally verbal children. Cochrane Database Syst Rev.

[27] Jurgens A, Anderson A, Moore DW (2019). Maintenance and generalization of skills acquired through picture exchange communication system (PECS) training: a long-term follow-up. Dev Neurorehabil.

[28] Alnazly EK, Abojedi A (2019). Psychological distress and perceived burden in caregivers of persons with Autism Spectrum Disorder. Perspect Psychiatr Care.

[29] Barros ALO, Gutierrez GM, Barros AO, Santos MTBR (2019). Quality of life and burden of caregivers of children and adolescents with disabilities. Spec Care Dentist.

[30] Singh P, Ghosh S, Nandi S (2017). Subjective Burden and Depression in mothers of children with Autism Spectrum Disorder in India: moderating effects of social support. J Autism Dev Disord.

