# Supplementary material for: Repercussion of the implementation of the Picture Exchange Communication System - PECS in the overload index of mothers of children with Autism Spectrum Disorder
Source: Codas. 2022 Jan 7;34(3):e20210109. doi: 10.1590/2317-1782/20212021109 (PMC9769430; doi:10.1590/2317-1782/20212021109)
Supplement: Translation

# Translation: Repercussão da implementação do *Picture Exchange Communication System – PECS* no índice de sobrecarga de mães de crianças com Transtorno do Espectro do Autismo

**Authors:** Carine Ferreira, Sheila Cavalcante Caetano, Jacy Perissinoto, Ana Carina Tamanaha

**Affiliations:** 1 Departamento de Fonoaudiologia, Universidade Federal de São Paulo – UNIFESP - São Paulo (SP), Brasil.; 2 Departamento de Psiquiatria, Universidade Federal de São Paulo – UNIFESP - São Paulo (SP), Brasil.

**Keywords:** Transtorno do Espectro do Autismo, Mães, Cuidadores, Comunicação, Fonoaudiologia

## Abstract

**Objetivo:**

O objetivo deste estudo foi analisar a repercussão da implementação do PECS no índice de sobrecarga de mães de crianças com Transtorno do Espectro do Autismo (TEA).

**Método:**

Trata-se de um estudo longitudinal (CEP 0403/2017). A amostra foi constituída por 20 díades mães-crianças com TEA. As mães tinham em média, 41 anos e 5 meses e as crianças, 7 anos e 2 meses. Quinze crianças eram do gênero masculino e cinco, do feminino. Foi aplicada a versão brasileira da escala *Burden Interview* cujo objetivo é a verificação do nível de sobrecarga dos cuidadores. Para análise dos comportamentos não-adaptativos aplicou-se o *Autism Behavior Checklist* (ABC) e para análise do repertório lexical: os Testes de Vocabulário Auditivo e Expressivo. O Programa de Implementação do PECS foi composto por 24 sessões de terapia fonoaudiológica individual com a presença ativa das mães. Ao final do Programa todas as crianças e suas respectivas mães foram reavaliadas com parte dos instrumentos utilizados na fase inicial do estudo.

**Resultados:**

Houve tendência de redução dos índices de sobrecarga materna após a implementação do PECS. Notamos diminuição significativa dos comportamentos não-adaptativos e aumento dos índices de vocabulário expressivo e auditivo das crianças no momento final do estudo. Não observamos correlação significativa entre os índices de sobrecarga com a idade, escolaridade e quociente intelectual das crianças; nem com a escolaridade e nível socioeconômico materno.

**Conclusão:**

Foi possível analisar a repercussão da implementação do PECS na sobrecarga de mães de crianças com TEA assistidas pelo Programa.

## INTRODUÇÃO

A sobrecarga de cuidadores de pessoas com patologias crônicas é descrita como uma perturbação resultante do lidar com a dependência física e a incapacidade mental do indivíduo alvo da atenção e dos cuidados. Ao assumir o papel de tutor, cuidador ou responsável pelo bem-estar e prestação de cuidados a um familiar dependente, o indivíduo fica sujeito à tensão e a agentes estressores, mas também a ganhos, tais como sentir satisfação e bem-estar pelo que pode proporcionar a seu familiar^(1)^.

O impacto inicial de um diagnóstico pode ser avassalador para as famílias sendo necessário um longo período de adaptação e equilíbrio. No caso do diagnóstico do Transtorno do Espectro do Autismo (TEA) essa realidade não é diferente.

O TEA caracteriza-se por impedimentos graves e crônicos nas áreas de interação e comunicação social, e por um repertório restrito e estereotipado de interesses^(2,3)^.

O comprometimento no desenvolvimento apresentado por crianças acometidas por essa condição pode ter diversas implicações para a dinâmica familiar, desde a sobrecarga física e mental decorrente de atribuições da vida cotidiana, altos níveis de estresse e baixo índice de qualidade de vida para seus familiares, até a possibilidade de se desenvolver a capacidade de adaptação e a resiliência. Estes aspectos podem interferir na vida pessoal, familiar, profissional e social; e podem predispor o cuidador a conflitos^(1,4-6)^.

Diante de uma condição tão invasiva e grave, a dinâmica familiar sofre mobilizações que vão desde aspectos relacionados à qualidade de vida física, psíquica, social e até financeira dos cuidadores diretos^(1,4-6)^. Os pais têm que fazer o luto pela perda do filho ideal, para que possam perceber as reais capacidades e potencialidades de sua criança^(6)^.

Estudos indicam que a adesão das famílias ao tratamento especializado tem impacto direto no desenvolvimento da criança e pode melhorar a dinâmica familiar com a redução da sobrecarga de todos os envolvidos^(1,3-6)^.

A atuação fonoaudiológica em linguagem com a criança com TEA pressupõe a participação da família, desde a avaliação à seleção e condução dos recursos de intervenção. Os sistemas de comunicação alternativos e aumentativos, como o *Picture Exchange Communication System* – PECS, fornecem um meio efetivo que permite que as crianças com habilidades de comunicação severamente limitadas exerçam controle sobre o meio ambiente solicitando itens preferidos^(7-12)^.

O treinamento dos pais na sua implementação tem apontado benefícios para crianças com TEA melhorando as respostas comunicativas delas. Outros estudos também relatam o aumento do número de vocalizações com intenção comunicativa ou a fala funcional, aquisições essas fundamentais para o desenvolvimento da comunicação humana^(9-12)^.

A hipótese que consideramos é a de que a participação orientada dos pais na intervenção fonoaudiológica com sistema de comunicação alternativa reduz a sobrecarga à medida que amplia os recursos de comunicação da criança.

O objetivo deste estudo foi analisar a repercussão da implementação do PECS no índice de sobrecarga de mães de crianças com TEA.

E como objetivos específicos: verificar o efeito do uso do PECS nos índices de comportamentos não-adaptativos e de vocabulário auditivo e expressivo. E por fim, correlacionar os índices inicial e final de sobrecarga materna à faixa etária, escolaridade, quociente intelectual das crianças, e à escolaridade e nível socioeconômico materno.

## MÉTODO

Trata-se de um estudo longitudinal.Todos os pais estiveram cientes dos procedimentos do estudo e assinaram o Termo de Consentimento Livre e Esclarecido, de acordo com as sugestões do Comitê de Ética em Pesquisa da Instituição (CEP 0403/2017).

### Amostra

Foi constituída uma amostra de conveniência, composta por 20 díades mães-crianças com TEA, atendidas no Núcleo de Investigação Fonoaudiológica de Linguagem da Criança e Adolescente no Transtorno do Espectro do Autismo – NIFLINC TEA do Departamento de Fonoaudiologia da Universidade Federal de São Paulo – UNIFESP, no período compreendido entre março de 2016 a março de 2019.

As mães tinham em média 41 anos e 5 meses (DP=7,9). Doze delas haviam completado ensino superior (60%) e oito (40%) ensino médio.

Em relação ao nível socioeconômico das famílias oito (40%) pertenciam a classe A/B (alta) e doze (60%) às classes C/D (média-baixa), de acordo com a classificação socioeconômica da ABEP^(13)^.

Do total de crianças, quinze eram do gênero masculino (75%) e cinco, do feminino (25%); na faixa etária entre 6 a 12 anos (média = 7 anos e 2 meses), atendidas e diagnosticadas com TEA por equipe multidisciplinar, segundo os critérios diagnósticos do DSM 5^(2)^.

Em relação à extensão de fala obtida a partir da aplicação da Avaliação do Comportamento Vocal^(14)^, 18 crianças (90%) demonstraram produção não verbal (emissão de balbucio e/ou vocalizações) e 2 crianças (10%), produção verbal mínima: emissão de palavras isoladas ou justaposição (com ausência de uso de verbos), durante o período de avaliação fonoaudiológica.

Em relação ao perfil cognitivo a distribuição dos valores de quociente intelectual se concentrou na faixa inferior, com pontuação média igual a 50,5 (DP= 9,4)^(15)^.

Todas as crianças estavam regularmente matriculadas em escolas de ensino regular devido à política brasileira de inclusão escolar, em média por quarenta e cinco meses (DP=21,9) e já haviam sido expostas à intervenção terapêutica fonoaudiológica prévia em serviços de assistência distintos, por no mínimo seis meses de duração, para garantia de que o perfil comunicativo fosse caracterizado como não verbal ou por verbalização mínima.

Como critérios de inclusão, consideramos: o diagnóstico de TEA, a faixa etária, a ausência de comunicação verbal ou verbalização mínima, a vinculação da criança em instituições educacionais e a disponibilidade da família em participar das sessões de terapia fonoaudiológica com adesão mínima de 75%.

Como critérios de exclusão, consideramos a presença de alterações neurológicas (acometimento estrutural e/ou funcional do Sistema Nervoso Central), malformações e/ou síndromes genéticas conhecidas, deficiências física, auditiva, visual e/ou motora.

### Procedimentos

Para avaliar a sobrecarga materna foi aplicada a versão brasileira da Escala *Burden Interview* ^(16)^ cujo objetivo é a verificação do nível de sobrecarga dos cuidadores de indivíduos com incapacidades mental e física, por meio de entrevista.

A escala inclui 22 perguntas que englobam as áreas de saúde, vida social e individual, situação financeira, estabilidade emocional e relações interpessoais. Essas perguntas refletem como as pessoas se sentem quando cuidam de outra pessoa. As questões referentes aos itens 1 ao 21 são pontuadas como: 0 - nunca; 1 - raramente; 2 - algumas vezes; 3 - frequentemente; 4 – sempre. A questão 22 é de caráter geral e nela o entrevistado é convidado a avaliar o quanto considera estar sobrecarregado devido ao seu papel. Nessa questão, as possíveis respostas correspondem: 0 - nem um pouco; 1- um pouco; 2 - moderadamente; 3 - muito e 4 - extremamente. Após todas as perguntas serem pontuadas e somadas, o escore final é obtido. Quanto maior o escore final, maior a sobrecarga do cuidador, sendo: < que 21 pontos - ausência de sobrecarga; de 21 a 40 pontos - sobrecarga moderada; 41 a 60 pontos - variação entre sobrecarga moderada a severa e > ou igual a 61 - sobrecarga severa.

Para análise dos comportamentos não-adaptativos foi aplicado, em forma de entrevista, o *Autism Behavior Checklist* (ABC)^(14)^, que consiste numa listagem de 57 comportamentos não adaptativos divididos em cinco áreas: sensorial, uso do corpo e objeto, relacional, linguagem e pessoal-social.

E para avaliação dos índices de vocabulário foram aplicados:

- Teste de Vocabulário Auditivo^(17)^, cujo objetivo é avaliar o vocabulário receptivo por meio de identificação de figuras;
- Teste de Vocabulário Expressivo^(17)^, cujo objetivo é avaliar o vocabulário expressivo da criança por meio de nomeação de figuras.

### O Programa de Implementação do PECS

O programa foi composto por 24 sessões de terapia fonoaudiológica individual com a presença das mães. Cada sessão teve duração de 45 minutos e foi realizada semanalmente. Todos os fonoaudiológicos envolvidos eram profissionais treinados e certificados no PECS. Todas as sessões foram filmadas para que os comportamentos das crianças fossem registrados nos protocolos de monitoramento dos progressos em cada fase, conforme proposto no Manual de Treinamento do PECS^(7)^. Os registros foram feitos por pesquisadores que não estiveram envolvidos no atendimento direto das crianças.

Após a finalização do Programa todas as crianças e suas respectivas mães foram reavaliadas com parte dos instrumentos utilizados na fase inicial do estudo.

### Método estatístico

Inicialmente foi realizada a análise descritiva dos dados. Aplicou-se o Teste T Pareado ou Wilcoxon para análise comparativa das variáveis: sobrecarga materna, índices do ABC e dos vocabulários auditivo e expressivo das crianças, nos dois momentos do estudo. Para análise das correlações entre os índices inicial e final de sobrecarga materna com: idade, escolaridade, quociente intelectual da criança; e a escolaridade e nível socioeconômico das mães, utilizou-se o Coeficiente de Correlação de Spearman. Considerou-se nível de significação de 0,05%.

## RESULTADOS

Na Tabela 1 podemos observar a frequência dos graus de sobrecarga materna nos dois momentos do estudo.

**Tabela 1:** Frequência dos graus de sobrecarga materna nos dois momentos

Momento /	Pré PECS		Pós PECS		
Grau de sobrecarga	N	%		N	%
Ausente	0	0%		1	5%
Leve	0	0%		0	0%
Moderada	12	60%		15	75%
Moderada Severa	6	30%		3	15%
Severa	2	10%		1	5%
Total	20	100%		20	100%

Legenda: N = número de mães

Na Tabela 2 podemos observar as comparações entre a sobrecarga materna, índices do ABC e dos vocabulários auditivo e expressivo das crianças, nos dois momentos do estudo.

**Tabela 2:** Análise comparativa entre a sobrecarga materna, índices do ABC e dos vocabulários auditivo e expressivo das crianças, nos dois momentos do estudo

		Pré	Pós	Teste t-pareado ou Wilcoxon (p)	Resultado
Sobrecarga	Média	43,0	39,0		
	Mediana	41,0	40,0	0,224	Pré = Pós
	Desvio-padrão	13,0	12,1		
	N	20	20		
ABC SE	Média	13,2	9,1		
	Mediana	13,5	9,5	0,001*	Pré > Pós
	Desvio-padrão	4,7	4,5		
	N	20	20		
ABC RE	Média	22,6	17,5		
	Mediana	22,0	18,5	<0,001*	Pré > Pós
	Desvio-padrão	6,8	6,4		
	N	20	20		
ABC CO	Média	20,0	16,8		
	Mediana	20,0	17,0	0,093*	Pré > Pós
	Desvio-padrão	7,9	7,8		
	N	20	20		
ABC LG	Média	13,3	10,5		
	Mediana	12,5	9,0	0,088	Pré = Pós
	Desvio-padrão	4,8	4,9		
	N	20	20		
ABC PS	Média	16,8	10,9		
	Mediana	18,5	10,5	<0,001*	Pré > Pós
	Desvio-padrão	4,1	5,2		
	N	20	20		
ABC Total	Média	85,9	64,0		
	Mediana	86,0	59,5	<0,001*	Pré > Pós
	Desvio-padrão	16,8	15,0		
	N	20	20		
Voc Auditivo	Média	3,0	7,4		
	Mediana	0,0	0,0	0,008*	Pré < Pós
	Desvio-padrão	8,0	12,6		
	N	20	20		
Voc Expressivo	Média	2,7	4,2		
	Mediana	0,0	0,0	0,018*	Pré < Pós
	Desvio-padrão	8,8	9,5		
		20	20		

* Significância estatística

Legenda: ABC SE = Autism Behavior Checklist Sensorial; ABC RE = Autism Behavior Checklist Relacional; ABC CO = Autism Behavior Checklist Uso do Corpo e Objeto; ABC LG = Autism Behavior Checklist Linguagem; ABC PS = Autism Behavior Checklist Pessoal Social; Voc = Vocabulário; N = Número de crianças

Na Tabela 3 podemos observar as correlações entre os índices inicial e final de sobrecarga materna com as variáveis idade, tempo de escolaridade e quociente intelectual da criança; e tempo de escolaridade materno e nível socioeconômico da família.

**Tabela 3:** Correlação entre os índices inicial e final de sobrecarga materna com as variáveis: idade, tempo de escolaridade e quociente intelectual da criança; tempo de escolaridade materna e nível socioeconômico

				Sobrecarga 1	Sobrecarga 2
Idade	Coeficiente Correlação			- 0,315	- 0,042
	P valor			0,176	0,860
	N			20	20
Escolaridade Criança	Coeficiente Correlação			0,392	- 0,124
	P valor			0,087	0,601
	N			20	20
QI	Coeficiente Correlação			- 0,164	- 0137
	P valor			0,491	0,566
	N			20	20
Escolaridade Materna	Coeficiente Correlação			0,002	- 0,267
	P valor			0,994	0,256
	N			20	20
NSE	Coeficiente Correlação			- 0,099	- 0,132
	P valor			0,688	0,589
	N			20	20

Legenda: N = número de crianças; QI = Quociente Intelectual; NSE = Nível socioeconômico

## DISCUSSÃO

Notamos que no momento inicial do estudo todas as mães referiram algum grau de sobrecarga, com predomínio de menção ao grau moderado. Esses resultados confirmam que as mães de crianças com TEA estão altamente vulneráveis à sobrecarga emocional e às situações de estresse parental, visto que os cuidados com o bem-estar dos filhos comumente recaem sobre elas^(1,3-6)^.

E embora não tenha havido significância estatística na análise comparativa dos índices médios de sobrecarga nos dois momentos do estudo (p=0,224), notamos uma tendência de redução dos valores após a implementação do Programa PECS. Houve discreta diminuição dos graus de sobrecarga severa, moderadamente-severa e menção a ausência de sobrecarga em um dos casos, no segundo momento do estudo. Esses resultados evidenciam a importância da participação ativa das famílias no tratamento^(18)^. É consenso que o empoderamento parental é fator preponderante para o enfrentamento dos desafios que o TEA impõe a todos os familiares^(18-22)^.

Outro ponto relevante que deve ser considerado aqui, refere-se à apropriação do sistema de comunicação alternativo e aumentativo que deve ter permitido trocas comunicativas mais eficientes entre as crianças e suas mães. Assim, ao incentivar e encorajar as crianças a demonstrarem seus desejos e intenções por meio dos cartões, o sistema PECS melhorou a qualidade da comunicação e consequentemente pode ter influenciado a redução da percepção de sobrecarga emocional das mães ao longo do programa^(9-12,18-22)^.

Na análise comparativa entre os dois momentos do estudo, verificamos que houve tendência de redução dos comportamentos não adaptativos observada em todas as áreas do ABC. Sendo que houve significância estatística nas áreas Sensorial (p=0,001), Relacional (p=0,001), Uso do Corpo e Objeto (p=0,093) e Pessoal-Social (0,001), bem como nos valores Totais (p=0,001). Essa diminuição de atipias comportamentais, observada pela perspectiva das mães, confirmou o impacto positivo do uso do PECS, não apenas possibilitando trocas comunicativas mais eficientes, mas também melhorando a própria qualidade das interações sociais. Mesmo na área da Linguagem (p=0,088) em que não houve significância estatística, o índice no segundo momento do estudo mostrou tendência de queda. Ao agregar pistas visuais à informação auditivo-verbal, o PECS ampliou a compreensão verbal e impactou positivamente as trocas comunicativas e o engajamento social^(9-12,23-27)^.

Observamos ainda, aumento significativo de respostas tanto na testagem do vocabulário expressivo (p=0,018), quanto auditivo (p=0,008). A melhora no desempenho comunicativo, tanto em seu caráter expressivo quanto receptivo, corroboram o efeito positivo do PECS no aumento de verbalização e sobretudo na compreensão verbal de seus usuários, conforme descrito por diferentes estudos^(7-12,23-27)^.

Não foram identificadas correlações significativas entre os índices inicial e final de sobrecarga materna e as variáveis idade (p=0,176; p=0,860), escolaridade (p=0,087; p=0,601) e quociente intelectual das crianças (p=0,491; p=0,566). Ou seja, tanto a faixa etária quanto o tempo médio de escolaridade e o quociente intelectual das crianças não influenciaram a percepção das mães sobre a sua sobrecarga. O mesmo ocorreu em relação ao tempo de escolaridade materno (p=0,994; p=0,256) e nível socioeconômico das famílias (p=0,688; p=0,589).

Portanto, a sobrecarga materna deve se vincular muito mais as preocupações inerentes à consciência de que o TEA é uma condição severa e vitalícia, que atinge profundamente às famílias independentemente da idade, potencial cognitivo ou fase de desenvolvimento em que a criança se encontra^(18-24)^.

E mesmo tendo ciência de que a condição financeira das famílias pode ser uma barreira para o acesso aos serviços especializados, o que pode acarretar o aumento do estresse parental^(21)^, neste estudo não verificamos correlação entre o nível socioeconômico e a sobrecarga materna. Talvez pelas famílias estarem vinculadas a um serviço de referência no tratamento do TEA, isto tenha amenizado a percepção de sobrecarga das mães nas entrevistas.

O cuidado em relação aos pais, ora proporcionando-lhes informações precisas sobre o desenvolvimento da criança, acolhendo as dúvidas e compreendendo pedidos, ora, convidando-os para participarem como agentes do processo da linguagem é tarefa fundamental no atendimento terapêutico fonoaudiológico da criança^(19)^.

Os resultados deste estudo nos conduzem à reflexão sobre a importância do olhar atento do fonoaudiólogo também para as famílias, principalmente para o cuidador principal. Pessoas com TEA demandam cuidados e sabendo do estado emocional deste cuidador, podemos melhorar e direcionar com mais precisão nossas condutas terapêuticas^(21-30)^.

### Limitações

Temos ciência de que o tamanho da amostra deve ser considerada uma limitação do estudo uma vez que pode ter reduzido o potencial do tratamento estatístico. Portanto, incentivamos a condução de mais estudos sobre os efeitos do PECS com amostras maiores, por períodos de exposição mais extensos e em ensaios clínicos com duplo-cegos. Além disso, recomendamos fortemente que estudos futuros considerem avaliar sintomas de ansiedade e depressão materna.

## CONCLUSÃO

Houve tendência de redução dos índices de sobrecarga materna após a implementação do PECS. Houve diminuição significativa dos comportamentos não-adaptativos e aumento dos índices de vocabulário expressivo e auditivo das crianças no momento final do estudo. Não observamos correlação significativa entre os graus de sobrecarga com a idade, escolaridade e quociente intelectual das crianças; nem com a escolaridade e nível socioeconômico materno.

Deste modo pudemos analisar a repercussão da implementação do PECS na sobrecarga de mães de crianças com TEA assistidas pelo Programa.
